# Risk factors for anesthesia-associated postoperative capillary leakage after thoracoscopic surgery in neonates: A single-center observational study

**DOI:** 10.3389/fped.2022.1051069

**Published:** 2023-01-04

**Authors:** Heqi Liu, Fang Wang, Jianmin Zhang, Zhengzheng Gao

**Affiliations:** Department of Anesthesiology, Beijing Children’s Hospital, Capital Medical University, National Center for Children’s Health, Beijing, China

**Keywords:** capillaries, neonates, postoperative complications, thoracoscopy, anesthesia

## Abstract

**Background:**

Thoracoscopy is considered the surgical method of choice for addressing a wide range of conditions in neonates. However, there is a lack of experience in anesthesia management for this procedure. On reviewing the newborns who had undergone thoracoscopic surgery at our medical center, some had developed edema after surgery. After excluding other etiologies, these neonates were diagnosed with capillary leakage secondary to thoracoscopy.

**Aims:**

This study aimed to identify the potential risk factors for capillary leakage secondary to thoracoscopy in neonates and to provide reference information for optimal anesthesia management.

**Methods:**

This single-center, retrospective, observational study examined neonates who had undergone thoracoscopic surgery between January 1, 2018, and September 31, 2021. Their electronic medical records were analyzed for demographic and clinical characteristics associated with anesthesia, and postoperative capillary leakage occurring within 24 and 48 h of surgery was assessed based on medical records.

**Results:**

A total of 56 neonates that underwent thoracoscopic surgery were included in this study. Postoperative capillary leakage within 24 h was diagnosed in 14 neonates (25%). The partial pressure of carbon dioxide was an independent factor influencing the occurrence of postoperative edema within 24 h (*P* = 0.021). Overall, 21 cases (37.5%) were diagnosed as postoperative capillary leakage within 48 h, and age was an independent factor influencing the occurrence of postoperative edema within 48 h (*P* = 0.027).

**Conclusions:**

According to our findings, we concluded that preventing the elevation of the partial pressure of carbon dioxide may reduce the occurrence of secondary capillary leakage within 24 h after thoracoscopic surgery, and that older newborns are less likely to have secondary capillary leakage within 48 h after thoracoscopic surgery. Our findings provide evidence that directly informs anesthesia management for thoracoscopic surgery in neonates.

**Clinical trial registration:**

The study was registered in the Chinese Clinical Trial Registry (ChiCTR2100054117).

## Clinical implications

### What is already known about the topic

• Neonatal thoracoscopic surgery causes little trauma, can provide a good surgical field, and can prevent the occurrence of chest wall malformations, chronic pain, and other short-term and long-term complications; however, there are few studies on anesthesia management within neonatal thoracoscopic surgery.
• Neonatal surgery, especially thoracoscopic surgery, is highly associated with hypercapnia. Although it is currently believed that hypercapnia does not affect cerebral oxygen saturation or hemodynamic stability and hence this hypercapnia seems to be admissible, there are lack of studies on the safety region for acceptable hypercapnia and hypercapnia.

### What new information this study adds

• Elevated partial pressure of carbon dioxide was an independent risk factor for secondary capillary leakage within 24 h after thoracoscopic surgery.
• Age was an independent risk factor for secondary capillary leakage 48 h after thoracoscopic surgery.

## Introduction

Neonatal thoracoscope surgery is conducive to observing the operative field, with the result of significantly reducing surgical complications. Moreover, due to the small amount of trauma caused by thoracoscopy, this surgical procedure can prevent future postoperative complications, such as chest wall deformities, scoliosis, and chronic pain in children. However, performance of neonatal thoracoscopic procedures is currently limited, especially in developing countries (1). This may be due to insufficient experience in perioperative anesthesia management (2) and a lack of research reports providing guidance and reference data.

The National Children's Medical Center of China at the Beijing Children's Hospital has carried out thoracoscopic surgery for almost four years. In doing so, we have paid close attention to the perioperative situation of the operated neonates. Unfortunately, we found pitting edema in some cases, mostly in the extremities, accompanied by clinical symptoms such as hypotension and hemoconcentration. We had consultation with a range of departments, including cardiology, nephrology, anesthesiology, and the neonatal intensive care unit, depending on the clinical manifestations [such as hypovolemic hypotension, reduced central venous pressure (CVP), little or no urine, decreased serum albumin, increased hemoglobin (Hb), and increased hematocrit (Hct)]; we ruled out cardiogenic edema, nephrogenic edema, and other possible differential diagnosis but ultimately diagnosed these neonates with capillary leakage secondary to surgery (3–5).

Capillary leakage has been used to describe a specific series of disease manifestations associated with increased capillary permeability to proteins. Diseases that can lead to capillary leakage include sepsis, idiopathic systemic capillary leak syndrome (SCLS), and Clarkson's disease (6). However, the above diseases were not found in the neonates with postural edema after thoracoscopic surgery, and these neonates did not develop acute kidney injury. Therefore, we considered that these neonates may have capillary leakage secondary to surgery. More specifically, we speculated that the cause of capillary leakage was not a specific clinical disease but may instead have occurred due to changes in perioperative factors caused by surgery and anesthesia.

This study aimed to discuss the possible influencing factors for capillary leakage occurring after surgery, including basic medical and demographic information as well as perioperative factors associated with anesthesia. Regardless of the cause, postoperative capillary leakage leads to a prolonged recovery time for these children, who are at risk of developing more serious complications, requiring a prolonged intensive care unit (ICU) stay, and incurring an increased financial burden on their families. Identifying possible risk factors is conducive to informing optimal thoracoscopic anesthesia management.

## Materials and methods

### Study preparation

We retrospectively examined the clinical data of neonates who had undergone thoracoscopic surgery at Beijing Children's Hospital (affiliated with the Capital Medical University, a National Center for Children's Health) between January 2018 and September 2021.

The study protocol complied with the Declaration of Helsinki and was approved by the Medical Ethics Committee at Beijing Children's Hospital [(2021)-E-210-R]. The study was registered in the Chinese Clinical Trial Registry (ChiCTR2100054117). The parents or legal guardians of all enrolled neonates provided their written informed consent for study participation.

### Data collection

**Figure 1:**
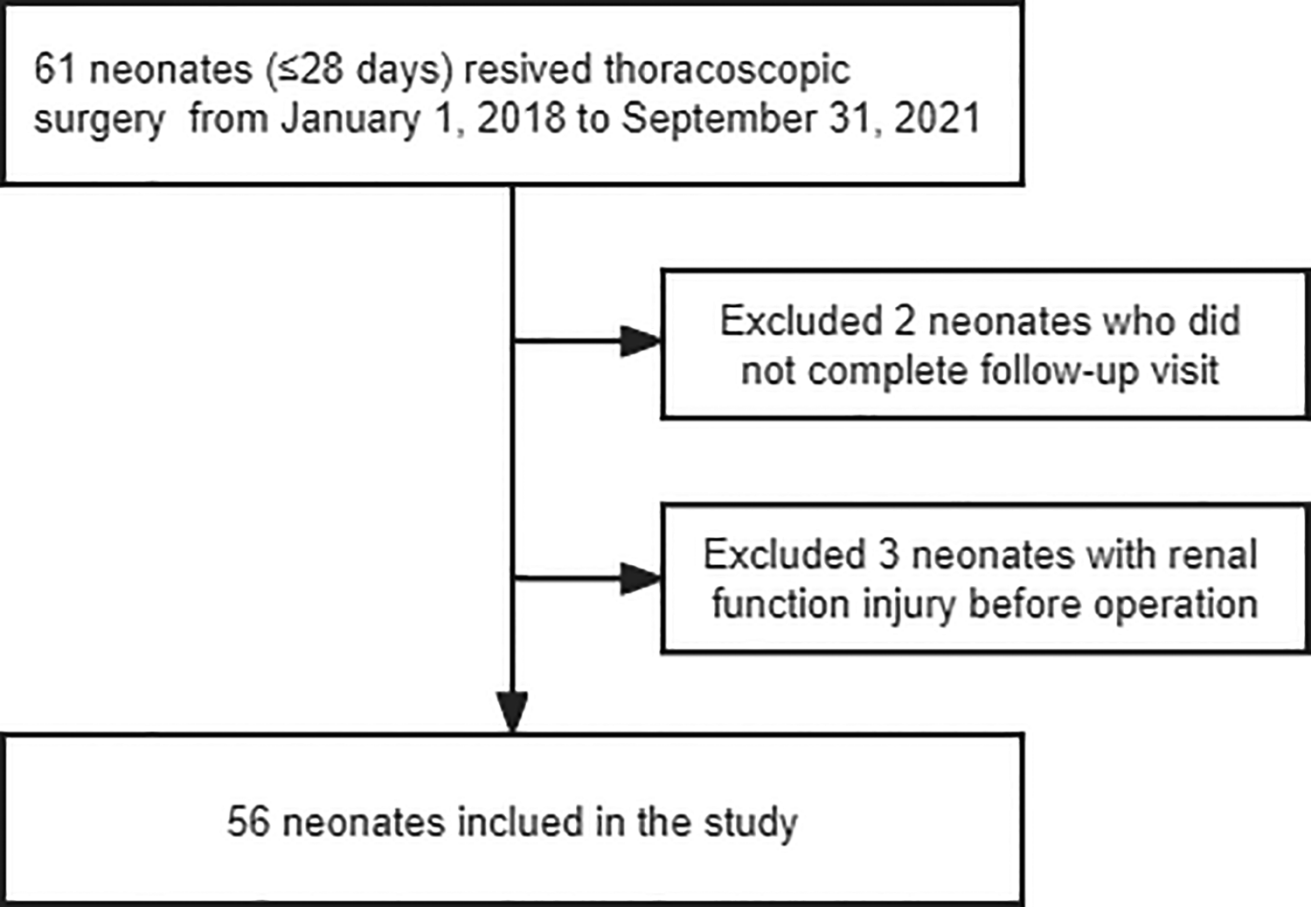
Study enrollment flowchart.

Electronic medical record and anesthesia information system databases (i.e., the JIAHE Medical Record System and the MEDICALSYSTEM DoCare Anesthesia Information System) were queried to obtain case data for all anesthetic exposures for neonates (aged ≤28 days) who underwent thoracoscopic surgery from January 1, 2018, to September 31, 2021. The patients were evaluated at follow-up visits at 24 and 48 h after the operation. Newborns who could not complete a follow-up visit and those who had edema or renal function injury before the operation were excluded from the current study (Figure 1). The authors performed a manual chart review for each patient included in the study. Data filtering and validation were performed to identify patients who met the clinical criteria for capillary leakage, as follows (3–5, 7):

Patients meeting the following four conditions were diagnosed with postoperative capillary leakage:

(1) Appearance of pitting edema/anasarca.
(2) Hypovolemic hypotension [mean arterial pressure <MAP> ≤gestational age in weeks, and urine volume <1 ml/kg or central venous pressure (CVP) ≤2 mmHg].
(3) A serum albumin concentration of ≤40 g/L, or a concentration that was decreased compared to the preoperative value, with Hb, Hct, and red blood cell counts remaining increased or unchanged in laboratory blood tests.
(4) Creatinine levels within the normal range for the corresponding age (8).

All neonates were subjected to a standardized anesthesia protocol. Monitoring during the operation was conducted using electrocardiography as well as blood pressure, peripheral oxygen saturation (SpO_2_), temperature, and end-tidal CO_2_ (EtCO_2_) readings. For the induction of anesthesia, 1–2 µg/kg fentanyl, 2–4 mg/kg propofol, and 0.1 mg/kg cis-atracurium or sevoflurane (3%–8% inspired concentration) was used along with a 30%–100% fraction of inspired oxygen. After intubated volume-controlled ventilation was applied (Vt [tidal volume]: 8–10 ml/kg, RR [respiratory rate]: 25–35 bpm [breaths per minute], I:E [inspiratory:expiratory ratio]: 1:1.5–1:1, FiO_2_ [fraction of inspired oxygen]: 0.8–1, PIP [peak inspiratory pressure]: 12–20 cmH_2_O, PEEP [positive end-expiratory pressure] 0–4 cmH_2_O), the ventilation was adjusted according to blood gas analysis and EtCO_2_ findings. One-lung ventilation was achieved through endobronchial intubation. It can be achieved with the aid of fiberoptic bronchoscope and railroading endotracheal tube into the desired mainstem bronchus. Anesthesia was maintained with 0–0.2 µg/kg/min remifentanil and sevoflurane.

Thoracoscopic surgery patients were placed on a heating mattress in a lateral position. The chest was insufflated with 6–8 mmHg CO_2_ at a flow rate of 4–6 L/min, depending on the surgical requirements; insufflation was stopped when the CO_2_ impeded adequate ventilation. The target heart rate was 10% above or below the resting heart rate, and the target MAP was >35 mmHg. Stable hemodynamics were achieved by fluid expansion and by regulating the depth of anesthesia. Pediatric electrolyte supplements injection was used for fluid expansion, 1000 ml of which contained 37.5 g glucose and 2.25 g sodium chloride. The infusion speed was adjusted to 10–20 ml/kg/h according to HR, MAP, and urine volume. After operation, all patients were transferred to neonatal intensive care unit (NICU) with endotracheal intubation. The physicians used midazolam for sedation (0.02–0.05 mg/kg/h). After the patient's circulation was stable and breathing has reached the extubation standard, tracheal extubation was performed and patients were transferred to the general ward. In NICU, balanced salt solution was used for liquid therapy.

Based on clinical experience and discussions with other physicians, we selected some factors that may presumably lead to capillary leakage for statistical testing. The demographic and basic medical data of interest was as follows: age, sex, weight, surgical procedure, premature birth status, disease (e.g., jaundice, congenital cardiac dysplasia, pneumonia).

Intraoperative conditions of interest included procedural duration, anesthesia time, intraoperative hypothermia time, heart rate, intraoperative hypotension time, one-lung ventilation, hypoxemia time, the use of steroids, the use of vasoactive agents, intraoperative fluid management, blood loss, and the results of intraoperative blood gas analysis.

We defined the procedural duration from the start of the skin incision to the end of skin suturing. Anesthesia time was defined as the time from the start of induction to the removal of the endotracheal intubation. The nasopharynx temperature of the operated newborns was monitored during the operation. We defined hypothermia as a temperature of ≤36°C (9–11). Heart rate was defined as the mean of heart rate measurements conducted at time points prior to induction, after intubation, at the start of surgery (i.e., skin resection), at the start of thoracoscopy, and at the end of surgery. Hypotension was defined as a 20% drop in the basal MAP, or a MAP that was less than that for the neonates' gestational age in weeks (12, 13). Depending on whether a child cries before induction, a basal blood pressure reading can be achieved at a quiet time. Hypoxemia time parameters were defined according to durations spent at peripheral oxygen saturations of ≤95% (including values ranging between 0% and 95%), ≤90%, and ≤80% (14). Intraoperative fluid management was performed with respect to expansion volume, colloid infusion volume, and urine volume.

Blood gas analysis was performed from arterial blood after removal of the thoracoscopic trocar. To facilitate statistical analysis, we selected several values of pH, carbon dioxide partial pressure (pCO_2_), base excess (BE), potassium (K^+^), and lactate (Lac) readings that may presumably be related to postoperative capillary leakage according to our clinical experience. Concurrently, to facilitate a more straightforward calculation procedure, we only recorded whether BE was within the normal range (±3).

### Statistical analysis

Categorical variables were presented as frequencies and percentages, and *χ*^2^ tests were used for inter-group comparisons. Continuous variables were tested for normality distribution using the Shapiro-Wilk test. Means ± standard deviations were calculated for normally distributed data, and independent sample t-tests were used for inter-group comparisons. Medians and interquartile ranges were used for non-normally distributed data, with Mann–Whitney *U* rank sum tests used for inter-group comparisons. Statistical significance was determined according to a two-sided *P*-value of <0.05.

We conducted a binary logistic regression analysis evaluating the influence of risk factors with a *P*-value <0.05 showing statistical differences at an *α* of 0.05. The Hosmere-Lemeshow goodness-of-fit test was used to examine the estimated-to-observed likelihood of outcomes.

## Results

From January 1, 2018, to September 31, 2021, 61 neonatal thoracoscopic surgeries (61 cases) were performed at our medical center, two of which were excluded because their parents/legal guardians did not follow medical advice and the patients were hence discharged after surgery for various reasons. In another three cases, we were unable to determine the cause of edema due to creatinine levels being higher than the normal index corresponding to the patient's age just prior to surgery. We finally included 56 neonates (Figure 1). The average age of these newborns was 6 (3,7) days and their mean weight was 3.03 ± 0.57 kg.

The operations performed in these patients included esophageal anastomosis and fistula repair (28/56, 50%), esophageal anastomosis (16/56, 28.6%), thoracic diaphragmatic repair (6/56, 16.1%), tracheoesophageal fistula repair (1/56, 2%), esophageal anastomosis and fundoplication (1/56, 2%), and a simple thoracoscopic examination (1/56, 2%); 14 cases (14/56, 25%) were diagnosed with postoperative capillary leakage within 24 h after surgery and 21 cases (21/56, 37.5%) were diagnosed with postoperative capillary leakage within 48 h after surgery through a careful review of their electronic medical records.

Demographic and clinical characteristics of the included participants are listed in Table 1. Within 24 h, there were no statistically significant differences in age, sex, weight, preterm birth, disease diagnoses (e.g., jaundice, congenital heart disease, and pneumonia), or surgical procedures between neonates who did or did not develop secondary capillary leakage. In addition, there were no statistically significant difference in fluid intake and fluid balance between fluid intake and output within 24 h after surgery. We detected statistically significant differences in pCO_2_ (*P* = 0.011) and Lac (*P* = 0.013) between patients with and without postoperative capillary leakage within 24 h of surgery.

**Table 1:** Demographic and clinic characteristics of the participants.

	24 h			48 h		
	no CL (*n* = 42)	CL (*n* = 14)	*P*-value	no CL (*n* = 35)	CL (*n* = 21)	*P*-value
Age (days)	4 (4,8)	5 (3,7)	0.886	5 (4,9)	4 (3,5)	0.009
Sex						
Female	13 (31.0%)	4 (28.6%)	1.000	9 (25.7%)	8 (38.1%)	0.329
Male	29 (69.0%)	10 (71.4%)		26 (74.3%)	13 (61.9%)	
Weight (kg)	3.04 ± 0.57	3.00 ± 0.62	0.821	3.15 (2.6,3.3)	2.85 (2.6,3.4)	0.980
Premature birth						
No	38 (90.5%)	11 (78.6%)	0.350	32 (91.4%)	17 (81.0%)	0.406
Yes	4 (9.5%)	3 (21.4%)		3 (8.6%)	4 (19.0%)	
Jaundice						
No	29 (69.0%)	10 (71.4%)	1.000	24 (68.57)	15 (71.4%)	0.822
Yes	13 (31.0%)	4 (28.6%)		11 (31.4%)	6 (28.6%)	
Congenital cardiac anomalies						
No	23 (54.8%)	7 (50.0%)	0.757	18 (51.4%)	12 (57.1%)	0.678
Yes	19 (45.2%)	7 (50.0%)		17 (48.6%)	9 (42.9%)	
Pneumonia						
No	19 (45.2%)	6 (42.9%)	0.877	16 (45.7%)	9 (42.9%)	0.835
Yes	23 (54.8%)	8 (57.1%)		19 (54.3%)	12 (57.1%)	
Surgical procedure						
EA	13 (31.0%)	3 (21.4%)	0.876	12 (34.3%)	4 (19.1%)	0.186
EA + TFR	19 (45.2%)	9 (64.3%)		13 (37.1%)	15 (71.4%)	
EA + F	1 (2.4%)	0 (0.0%)		1 (2.9%)	0 (0.0%)	
TFR	1 (2.4%)	0 (0.0%)		1 (2.9%)	0 (0.0%)	
DR	7 (16.7%)	2 (14.3%)		7 (20.0%)	2 (9.5%)	
ST	1 (2.4%)	0 (0.0%)		1 (2.9%)	0 (0.0%)	
Procedural duration (min)	96.9 ± 24.6	106.8 ± 36.1	0.253	95.4 ± 28.1	106.0 ± 26.9	0.172
Anesthesia time (min)	154.2 ± 32.4	169.7 ± 45.2	0.168	152.1 ± 37.6	168.1 ± 32.2	0.108
One-lung ventilation						
No	36 (85.7%)	12 (85.7%)	1.000	31 (88.6%)	17 (81.0%)	0.456
Yes	6 (14.3%)	2 (14.3%)		4 (11.4%)	4 (19.1%)	
Use of vasoactive drugs						
No	38 (90.5%)	11 (78.6%)	0.350	31 (88.6%)	18 (85.7%)	1.000
Yes	4 (9.5%)	3 (21.4%)		4 (11.4%)	3 (14.3%)	
Use of steroid hormone						
No	16 (38.1%)	8 (57.1%)	0.212	13 (37.1%)	11 (52.4%)	0.265
Yes	26 (61.9%)	6 (42.9%)		22 (62.9%)	10 (47.6%)	
Hypothermia time (min)	50 (0,80)	35 (0,90)	0.751	30 (0,70)	80 (0,90)	0.225
Heart rate (bpm)	142.4 ± 15.9	137.9 ± 16.1	0.362	142.0 ± 16.4	140.0 ± 15.4	0.653
Hypotension time (min)	20 (5,55)	40 (5,70)	0.215	20 (5,55)	20 (5,60)	0.708
Hypoxemia time (min)						
≤95%	45 (10,80)	25 (10,70)	0.424	30 (10,80)	40 (20,70)	0.753
≤90%	10 (0,20)	10 (0,20)	0.497	10 (0,20)	10 (0,20)	0.799
≤80%	0 (0,10)	0 (0,10)	0.826	0 (0,10)	0 (0,10)	0.835
Expansion volume (mL/kg)	25.2 (18.3,30.8)	17.9 (12.7,54.9)	0.226	24.7 (17.1,30.5)	25.0 (16.4,42.5)	0.565
Colloid infusion volume (mL/kg)	0 (0,0)	0 (0,0)	0.564	0 (0,0)	0 (0,0)	0.439
Urine volume (mL/kg)	3.0 (1.6,4.3)	2.7 (1.7,3.7)	0.583	3.0 (1.6,3.8)	2.9 (1.6,4.1)	0.919
Blood loss (mL/kg)	0.4 (0.3,0.6)	0.6 (0.4,0.7)	0.179	0.4 (0.3,0.6)	0.4 (0.3,0.7)	0.624
pH	7.34 (7.3,7.38)	7.33 (7.29,7.44)	0.677	7.33 ± 0.10	7.35 ± 0.09	0.353
pCO_2_ (mmHg)	41.2 ± 11.9	52.4 ± 18.4	0.011	45.9 ± 13.6	40.94 ± 15.76	0.218
K + (mmol/L)	3.6 (3.2,3.8)	3.8 (3,4)	0.476	3.8 (3.2,4)	3.3 (3,3.8)	0.133
Lac (mmol/L)	0.8 (0.4,1.1)	1.1 (0.9,2.1)	0.013	0.8 (0.4,1.2)	0.9 (0.8,1.6)	0.228
BE						
Normal	35 (83.3%)	11 (78.6%)	0.698	29 (82.9%)	17 (81.0%)	1.000
Abnormal	7 (16.6%)	3 (21.4%)		6 (17.1%)	4 (19.1%)	
Fluid intake in first 24 h (ml/kg)	130.0 ± 35.9	118.5 ± 34.3	0.629			
Fluid balance at 24 h (ml/kg)	54.1 ± 45.4	45.1 ± 35.8	0.503			
Fluid intake in 24-48 h (ml/kg)				119.2 ± 24.6	109.3 ± 21.1	0.659
Fluid balance at 24-48 h (ml/kg)				34.0 ± 32.5	42.9 ± 30.2	0.384
Fluid intake in 48 h (ml/kg)				247.5 ± 38.8	236.4 ± 56.1	0.244
Fluid balance at 48 h (ml/kg)				82.3 ± 59.7	100.4 ± 53.8	0.695

BE, base excess; CL, capillary leakage; DR, thoracoscopic diaphragmatic repair; EA, esophageal anastomosis; F, fundoplication; K^+^, potassium; Lac, lactate; pCO_2_, partial pressure of carbon dioxide; ST, simple thoracoscopic; TFR, tracheoesophageal fistula repair.

Binary logistic regression was conducted with pCO_2_ and Lac as independent variables; whether postoperative capillary leakage occurred within 24 h was considered a dependent variable. Our results showed that pCO_2_ was an independent factor influencing the occurrence of postoperative capillary leakage within 24 h (OR, 1.057; 95% CI, 1.008–1.108; *P* = 0.021). The Hosmer-Lemeshow goodness-of-fit test was non-significant (*P* = 0.157), indicating that the model exhibited a good fit. Moreover, we found a statistically significant difference in the age (*P* = 0.009) of patients with and without postoperative capillary leakage occurring within 48 h. Moreover, binary logistic regression revealed that age was an independent factor influencing the occurrence of postoperative capillary leakage within 48 h (OR, 0.737; 95% CI, 0.562–0.966; *P* = 0.027) (Table 2).

**Table 2:** Binary logistic regression for factors affecting postoperative capillary leakage.

	*β*	*P*	OR	95% CI
Factors within 24 h				
pCO_2_	0.055	0.021	1.057	1.008–1.108
Lac	0.310	0.252	1.363	0.802–2.317
Factor within 48 h				
Age	−0.305	0.027	0.737	0.562–0.966

CI, confidence interval; Lac, lactate; OR, odds ratio; pCO_2_, partial pressure of carbon dioxide.

## Discussion

This study determined that, at our medical center, the occurrence rates of capillary leakage secondary to surgery were 25% within 24 h after surgery and 37.5% within 48 h after surgery. Moreover, we reviewed the electronic medical record information and anesthesia record sheets of the included children to determine risk factors related to anesthesia that may lead to the capillary leakage. The results presented in Table 2 show that pCO_2_ was an independent factor influencing the occurrence of postoperative capillary leakage within 24 h after surgery. In addition, under the condition of other factors being unchanged, the ratio of occurrence and non-occurrence of postoperative capillary leakage within 24 h was increased 1.057-fold given a pCO_2_ increase of 1 mmHg. Age was also an independent factor influencing the occurrence of postoperative capillary leakage within 48 h. With other conditions unchanged, the ratio of occurrence and non-occurrence of postoperative capillary leakage within 48 h decreased 0.737-fold for each day of increased neonate age.

All diseases causing capillary leakage syndrome share a common pathophysiological abnormality; that is, increased capillary permeability to proteins (7). The utility of the endothelium as a barrier between the intravascular and interstitial spaces depends on the integrity of binding between adjacent endothelial cells (15). When the bonding between endothelial cells is broken, the barrier capacity is impaired and protein-rich fluid is lost from the blood vessels into the interstitial space (16). Although changes in endothelial cell connectivity have not been studied with regard to certain causes of capillary leakage syndrome, the common clinical phenotypes in these diseases suggest similar molecular pathophysiology (6).

Concurrently, in terms of hemodynamics, loss of protein-rich fluid from the intravascular space results in intravascular volume decrease, leading to secondary activation of the renin, angiotensin, and aldosterone system and the sympathetic nervous system as well as the release of vasopressin. Sodium and water retention lead to systemic edema and exudative serous cavities (17, 18). The depression edema in neonates after thoracoscopic surgery observed in our study cannot be explained by other reasons (which may share similar molecular pathophysiology). We noted that the breaking of the binding between endothelial cells leads to protein leakage, and edema may be a manifestation of capillary leakage occurring secondary to thoracoscopic surgery.

Hypercytokinemia is believed to be the underlying cause of capillary leakage (16, 19). Cytokines have been shown to increase vascular permeability by disrupting the adherent junctions (20, 21). During the capillary leak phase, a soluble factor in the plasma increases endothelial permeability, thereby leading to capillary leakage.

In our study, elevated pCO_2_ in blood gas analysis was an independent risk factor for capillary leakage within 24 h after surgery. As mentioned above, the mechanism of capillary permeability changes may be mediated by cytokine release. In parallel, studies at the cell level have demonstrated the profound effect of CO_2_ on multiple diverse signaling pathways, be it effects from CO_2_ itself or from the associated acidosis it generates. In an experiment in adult rats, hypercapnia-induced IL-1*β* (interleukin-1 beta) overproduction in the hypoxemic blood was found to decrease tight junctional protein expression in cerebrovascular endothelial cells *via* the IL-1R1/p-IRAK-1 (interleukin receptor/interleukin receptor kinase) pathway, eventually resulting in increased blood-brain barrier permeability (22). Therefore, we conclude that elevated pCO_2_ may affect the connection of capillary endothelial cells through the induction of cytokines. The specific mechanisms mediating the relationship between cytokine release and secondary capillary leakage require further study.

During the past decade, thoracoscopic surgery has made significant advances (23). This surgical method can provide excellent results with regard to technical aspects (working space, anatomy, complications) (24), and is considered the surgical method of choice for a wide range of conditions in neonates and infants (as opposed to standard thoracotomy). However, there is a paucity of information on the relevant intraoperative physiology during the neonatal period and the corresponding management schemes and concepts in anesthesia have not yet been developed (23, 25, 26). Moreover, thoracoscopic surgery presents a larger physiological challenge for newborns and younger children (26, 27). Recent evidence suggests that severe intraoperative acidosis and hypercapnia may occur during advanced thoracoscopic surgery. Some studies have found that neonates undergoing operative repair of congenital diaphragmatic hernia and esophageal atresia/tracheoesophageal fistula develop intraoperative acidosis and hypercapnia, wherein the rate of absorption of CO_2_ during laparoscopy (approximately 20%) seems to be smaller than that during thoracoscopy (28). Insufflated carbon dioxide passes across the pleural membrane to be absorbed into the bloodstream. Raised intrathoracic pressure impairs ventilation, leading to hypercapnia (29–31). Some surgeons prefer CO_2_ injection during thoracoscopic repair to create a workspace, as it is non-combustible, inexpensive, and unlikely to cause embolism (30).

As described above, reversible hypercapnia can occur during thoracoscopic surgery in children, especially neonates; this is consistent with our findings in this retrospective study. In the blood gas analysis of newborns after thoracoscopic surgery, acidemia was found to occur in 60.7% (34/56) of patients, whereas hypercapnia occurred in 41.1% (23/56). There are reports that neonatal cases of congenital diaphragmatic hernia can tolerate thoracoscopic surgery, despite transient reversible deterioration in the acid/base balance (32). A cohort study also suggested that thoracoscopic repair of congenital diaphragmatic hernia (CDH) and esophageal atresia (EA) using artificial CO_2_-pneumothorax (aims to create a modified surgical field) leads to severe acidosis in neonates. However, regional cerebral oxygen saturation (rSO_2_) remained within clinically acceptable limits during periods of acidosis. Neurodevelopmental outcomes were also favorable within the first 24 months in a prior study, although long-term outcomes in this specific patient group deserve further investigation (33).

In our study, elevated pCO_2_ was found to increase the risk of secondary capillary leakage in neonates. Capillary leakage occurred in 43.4% (10/23) of newborns with hypercapnia within 24 h after operation. We conclude that the admissibility of hypercapnia may be due to insufficient research. In the above studies, hypercapnia was reversible. Moreover, hypercapnia may not cause pathological changes during surgery. However, it cannot be excluded that this condition may be associated with some postoperative complications. Moreover, the reversibility of hypercapnia may lead anesthetists to lose sight of necessary vigilance in conducting this procedure.

There is currently no consensus on optimal pCO_2_ levels and the effectiveness of permissive hypercapnia in newborns (34). The UK National Institute of Health and Clinical Excellence (NICE) guidelines support the use of thoracoscopy for the repair of CDH in neonates, but do not address safety concerns related to hypercapnia and acidosis. However, these guidelines encourage collection of data and publication of results on this procedure. In future studies, we plan to focus on ventilation strategies with a focus on thoracoscopic surgeries in neonates. A more targeted anesthesia protocol needs to be established.

This study also observed that younger neonatal age was an independent risk factor for capillary leakage occurring within 48 h after surgery. A study in children undergoing open heart surgery found that smaller patient size (height and weight), younger age, and longer cardiopulmonary bypass (CPB) time were incremental risk factors for a rise in total body water (35). As the timing of neonatal thoracoscopic surgery may be considered in relation to many aspects, we cannot simply demand that children have their operation postponed from the perspective of reducing perioperative complications. The primary disease in the presenting newborns is the main factor in the selection of the operation time. However, for some elective operations, we suggest delaying the operation and waiting for the children to mature.

Moreover, if children have to undergo thoracoscopic surgery at a younger age, the occurrence of postoperative capillary leakage should be vigilantly monitored. For example, pCO_2_ was determined to be an independent risk factor within 24 h after surgery. However, pCO_2_ did not show a positive result in the 48 h risk factor analysis. This may be because capillary leakage within 48 h after operation was more affected by postoperative treatment. Physicians in the NICU often first supplement with a balanced salt solution to maintain perfusion after identifying postoperatively hypotensive newborns; the child then continues mechanical ventilation in the PICU until the surgeon assesses that the child's condition is stable, and no further restriction of activity is required. The endotracheal intubation is then removed in combination with an assessment of the child's respiratory function. We noted that the duration of endotracheal intubation varies greatly among newborns. All the above may affect capillary leakage 48 h after surgery. Hence, in the future, we need the cooperation of doctors in the NICU in order to review a larger number and greater range of postoperative treatment records with the goal of informing more optimal surgical anesthesia management.

Although no statistically significant relationship was found between intraoperative steroid use and postoperative capillary leakage in our retrospective study, steroid therapy has demonstrated efficacy in capillary leakage occurring due to medications exposure, differentiation syndrome, engraftment syndrome, and autoimmune diseases. Since capillary leakage is generally considered a cytokine mediated disease, the benefits of steroids may be related to their ability to reduce the expression of multiple cytokines (36).

The current investigation was an observational study enrolling a small number of cases. In addition, we were informed by our clinical practice experience at our medical center. Anesthesiologists tend to have strong personal preferences for steroid use in clinical practice, though the dosage range used somewhat differs. Therefore, we believe that the relationship between steroid hormones and capillary leakage after neonatal thoracoscopic surgery cannot fully inform definitive conclusions within our retrospective study. A prospective cohort study with a rigorous experimental design is needed to confirm these findings more definitively.

When treating patients with postoperative capillary leakage, the protocol at our medical center first involves evaluating the child's circulation volume and then considers the early use of diuretics to relieve edema along with enhancement of colloid rehydration therapy. For some children with severe symptoms, plasma infusion is also used to increase osmotic pressure. In general, edema symptoms gradually resolve, and these symptoms improve after an average of 3 (0,6) days.

Although the long-term complications of postoperative capillary leakage have not been documented in rigorous research, the occurrence of postoperative capillary leakage is known to prolong pediatric patients' ICU and general hospital stays and to increase the economic burden on children's families based on substantial clinical experience. We noted that our study findings are provisional and await confirmation within larger, prospective studies.

Since this was a single-center retrospective study, our results were limited in many respects. First, all data were derived from medical records, and we could not rule out recording deviations and human error. To reduce human error, we chose more objective data or data collected directly from the computer and monitor whenever possible. However, on the other hand, blood pressure, sPO_2_, and temperature are recorded every 5 min in the anesthesia information system, and the data collected may differ from the actual situation. Second, the sample size of this study was limited. In future studies, we will work with other pediatric surgery centers in order to recommend a neonatal thoracoscopic anesthesia procedure based on more comprehensive evidence. Third, we could not categorize severity except with regard to the duration of capillary leakage. Cordemans et al. previously used the capillary leakage index (CLI = CRP (mg/ DL)/serum albumin (g/L) *100) to calculate the severity of sepia-induced CL (37). This may not be applicable to the CL secondary to surgery discussed in this study. Therefore, it is necessary to determine a new, comprehensively validated severity evaluation indicator.

## Conclusions

Elevated pCO_2_ is a risk factor for secondary capillary leakage occurring within 24 h after surgery. Therefore, prevention of pCO_2_ increases in neonates undergoing thoracoscopic surgery is a necessary goal within surgical anesthesia management. Moreover, age is a risk factor for secondary capillary leakage within 48 h. Choosing the optimal time to schedule the operation (according to a range of factors) is beneficial for preventing the occurrence of postoperative capillary leakage. More clinical studies and guidelines are needed to improve the currently practiced anesthesia regimen for neonatal thoracoscopic surgery in general and to help less-experienced medical centers perform neonatal thoracoscopic surgery.

## Data Availability

The original contributions presented in the study are included in the article/Supplementary Materials, further inquiries can be directed to the corresponding authors.
